# Ketamine Protocol for Palliative Care in Cancer Patients With Refractory Pain

**Published:** 2015-11-01

**Authors:** Brighton A. Loveday, Jill Sindt

**Affiliations:** Huntsman Cancer Hospital, Salt Lake City, Utah

Pain is a common complication for cancer patients, with an incidence as high as 90% of those with advanced cancer (Lossignol, Obiols-Portis, & Body, 2005). Intractable or refractory pain has been reported to occur in 10% to 20% of patients with cancer and is difficult to control with opioids alone (Afsharimani, Kindl, Good, & Hardy, 2015).

Refractory cancer pain is often due to disease progression and leads to exacerbation of preexisting pain and/or complex pain syndromes, including neuropathic pain or plexopathy. The development of both chronic pain and intractable cancer pain appears to be related to the activation of N-methyl-D-aspartate (NMDA) receptors in certain regions of the central nervous system, especially in the dorsal horn, the limbic system, and the corpus callosum (Lossignol, Obiols-Portis, & Body, 2005).

Ketamine is an NMDA antagonist anesthetic agent. Although it can be used in high doses to induce general anesthesia, it is more often used outside the operating room at subanesthetic doses as a coanalgesic, usually in combination with opioids. It appears to be particularly useful for the treatment of neuropathic pain and is the most potent NMDA-receptor channel blocker available for clinical use (Afsharimani, Kindl, Good, & Hardy, 2015; Quibell, Prommer, Mihalyo, Twycross, & Wilcock, 2011). Although ketamine has been used in palliative care, evidence has varied regarding its efficacy. Additionally, there is a great variance in practice regarding the dose, route, and frequency of delivery.

At our institution, our supportive oncology and survivorship team staffs a regional, free-standing, and university-affiliated cancer hospital. Providers on our supportive oncology and survivorship team include physicians specializing in anesthesiology and pain medicine, psychiatry, oncology and palliative care, and nurse practitioners with specialty training in pain and palliative care as well as oncology. The scope of care often includes cancer patients who are admitted to the hospital in pain crises, with varying functional levels and symptom burden.

In this article, we share our guidelines for the use of subanesthetic ketamine infusions when escalating opioids and adjuvant analgesics have proved to be ineffective for intractable pain alone. We also review two case scenarios in which we found ketamine infusions to be particularly helpful.

## MECHANISM OF ACTION

Originally developed in 1962, ketamine is a highly lipophilic and water-soluble anesthetic agent. It is a phencyclidine (PCP) derivative that easily crosses the blood-brain barrier and is metabolized via cytochrome P450 isoenzyme 3A4. Its primary mechanism in producing anesthesia and analgesia is thought to be via noncompetitive NMDA-receptor antagonism, although it has activity at multiple receptors (Prommer, 2012).

In subanesthetic doses, ketamine allows the patient to remain awake and responsive while producing significant analgesia. It rarely causes respiratory depression at low doses and does not depress cardiovascular function, hepatic blood flow, or bowel function (Pasero & McCaffery, 2005). In addition, subanesthetic doses have minimal impact on heart rate or blood pressure, making it safe to use in patients with hemodynamic instability. The IV onset of action is within seconds, and the half-life is 2 to 3 hours (Prommer, 2012).

Ketamine is considered one of the World Health Organization’s essential drugs for the management of refractory pain and is associated with reduced opioid consumption and reduced opioid tolerance (Prommer, 2012). Its analgesic mechanism at the NMDA receptor is thought to be secondary to antineuropathic activity, as well as the evolving understanding of potentiation of opioids via reduced opioid tolerance (Salas et al., 2012).

The NMDA receptors are located throughout the nervous system and mirror the location of opioid receptors. There is an association among nociceptive activation of the NMDA receptor, clinical hyperalgesia or allodynia, and reduced opioid sensitivity. There is growing evidence suggesting that the NMDA receptor plays an active role in the development of opioid tolerance (Kerr, Holahan, & Milch, 2011; Bell, Eccleston, & Kalso, 2012). Thus, NMDA-receptor antagonists such as ketamine may reduce opioid tolerance by blocking the glutamate-induced activation of the NMDA receptor (Bell et al., 2012).

In our practice, we have found that ketamine improves patients’ response to opioids and reduces the overall opioid requirements required to control severe cancer-associated pain.

## POSSIBLE SIDE EFFECTS

Side effects of ketamine are primarily dissociative in origin, including dysphoria, misperceptions, hallucinations, and alterations in body image or mood. Although these symptoms occur minimally with subanesthetic analgesic doses, concerns about potential side effects frequently and needlessly limit its use.

Subanesthetic doses are more commonly associated with mild and transient impairments in attention, memory, and judgment (Quibell, Prommer, Mihalyo, Twycross, & Wilcock, 2011; Winegarden, Carr, & Bradshaw, 2015). If they occur, these effects can generally be controlled by concurrent administration of low doses of benzodiazepine or haloperidol, such as lorazepam 0.5 to 1 mg. Mild increases in heart rate or blood pressure can also occur. Neither the analgesic effect of ketamine nor potential side effects are reversed by naloxone.

## USE IN PAIN MANAGEMENT AND PALLIATIVE CARE

A majority of the evidence for ketamine in the treatment of pain is centered on its use in the perioperative period, as a strategy to reduce postoperative pain and postoperative opioid consumption. Studies consistently show a benefit in this setting (Bell, Dahl, Moore, & Kalso, 2006).

A Cochrane review updated in 2012 studying the use of ketamine for cancer pain included two randomized controlled trials that evaluated the effectiveness of adding ketamine to opioids in cancer patients with refractory pain (Afsharimani, Kindl, Good, & Hardy, 2015). Both studies found that ketamine administered intravenously or intrathecally was effective in improving analgesia.

However, given the small number of patients and the clinical heterogeneity in these studies, the evidence was insufficient to assess the benefits and harms of ketamine for the treatment of cancer pain (Afsharimani, Kindl, Good, & Hardy, 2015). Most of the evidence supporting ketamine’s efficacy as an analgesic for cancer pain is from case reports, retrospective surveys, or uncontrolled studies in patients with refractory neuropathic, bone, and mucositis-related pain (Quibell et al., 2011; Winegarden et al., 2015).

Ketamine is generally used in addition to or as an alternative to morphine. In our experience, we combine it in a continuous infusion with IV morphine, hydromorphone, or fentanyl for optimal effect. Our criteria for considering the addition of subanesthetic doses of ketamine include situations when intractable cancer pain has not responded to increases in opioids or when opioid escalation is precluded by undesirable or dose-limiting side effects.

## DEVELOPMENT OF A KETAMINE PROTOCOL

The ketamine protocol in use at our facility was originally developed in 2008 by the Department of Anesthesiology for use by the Acute Pain Service at the university hospital affiliated with, and physically connected to, our cancer hospital. The Acute Pain Service manages patients with complicated or difficult-to-control pain, primarily in the postoperative setting. They employ both medication management and appropriate acute pain interventions such as thoracic epidurals and peripheral nerve blocks and catheters.

The protocol was developed in response to patients on chronic opioids whose acute pain was not well managed with conventional methods. It also was formed with an eye toward safety, given the potentially large doses of opioids required to control these patients’ pain and the attendant risks of serious side effects such as sedation and respiratory depression.

The protocol was written after an extensive review of the literature regarding the use of subanesthetic ketamine for the management of acute pain. Weight-based dosing was chosen, as the research consistently showed minimal (if any) side effects using ketamine doses of less than 0.2 mg/kg/hr. A continuous infusion was chosen instead of use in a patient-controlled analgesia (PCA) device, also for a reduced risk of side effects. The protocol was presented and approved by the hospital Pharmacy and Therapeutics Committee for use by only specialized pain services at the Institution including the Acute Pain Service and the Supportive Oncology Service.

Following protocol approval, extensive education about ketamine was provided, with regular presentations to nursing and pharmacy staff. The biggest concern raised by the nursing staff about using ketamine was unfamiliarity with it and the risk of side effects, including hallucinations, dysphoria, tachycardia, and hypertension. Increased availability and response by the Acute Pain Service for questions and education were offered, and patient experience with the ketamine protocol was closely monitored. In patients receiving ketamine infusions, a very low incidence of side effects was noted, pain control was improved, and opioid use was decreased. Given the success in managing acute postoperative pain, the ketamine infusion has been expanded for use in the palliative care setting for acute cancer pain, with continued success.

**Ketamine Protocol Details**

The ketamine infusion is available only to specialized pain services at our institution. Patients are seen and evaluated for the presence of intractable cancer pain unresponsive to opioid escalation or limited in opioid escalation due to intolerable side effects. Use of other adjunct medications including nonsteroidal anti-inflammatory drugs (NSAIDs), steroids, neuropathic medications, and acetaminophen is considered, as well as procedural interventions such as nerve blocks, intrathecal medication delivery, and radiation therapy.

The protocol is composed of a weight-based continuous infusion, starting at 0.1 mg/kg/hr with a maximum infusion rate of 0.2 mg/kg/hr or 20 mg/kg/hr, whichever is lower. Administration instructions include orders for the nursing staff to adjust the dose to patient comfort without side effects; the dose is typically increased by 2 mg/hr every 2 hours for inadequate pain control to a maximum of 20 mg/hr.

Communication orders include notifying the Pain Service in the event of the following: (1) abnormal hemodynamic parameters (blood pressure greater than 160/100 mm Hg, heart rate >110 beats per minute, or respiratory rate less than or equal to 8 breaths per minute); (2) inadequate control of pain, pruritus, nausea, or vomiting; and (3) signs of hallucinations, dysphoria, or severe agitation. Additional orders are included to suspend other systemic opioids or central nervous system depressants, except those written by the Pain Service. The protocol also includes continuous pulse oximetry and low-flow oxygen therapy per nasal cannula as needed to achieve a pulse oximetry greater than 90%.

The aim of the ketamine infusion is to initially interrupt the pain crisis by increasing the ketamine infusion rate incrementally until the patient is comfortable. Next, the pain crisis is reversed by continuation of the hourly ketamine dose for approximately 24 hours followed by gradual dose reduction as tolerated. After the dose has been reduced to less than 10 mg/hr, the ketamine infusion can be safely discontinued without risk of worsened pain or other side effects.

Once the pain crisis has improved using the ketamine infusion, patients with cancer pain are likely to continue to require opioid and adjuvant pain medications, although with improved pain control and overall reduced doses allowing gradual taper and transition to oral and/or transdermal pain regimens. Occasionally, this transition to oral and transdermal pain medications is not feasible due to dysphagia, gastrointestinal tract or subcutaneous absorption, allergy, or other concerns. In these cases, we consider placing an intrathecal pump for long-term pain management. These pumps are implanted by a spine surgeon or anesthesiologist trained in interventional pain management.

When patients with cancer pain are discharged from our hospital, they are offered and scheduled for follow-up with our Supportive Oncology and Survivorship service in the outpatient setting for continued pain and symptom management in the setting of palliative care.

## APPROPRIATE USE OF KETAMINE INFUSIONS

Continuous ketamine infusions at subanesthetic doses are considered appropriate for cancer patients in the hospital when pain is not controlled despite escalating doses of medications or when the dose-limiting side effects prevent further dose escalation. A ketamine infusion of this type functions ideally in the setting of complex pain syndromes involving both nociceptive and neuropathic contributors that are clearly refractory to opioid escalation and the addition of adjuvant medications such as tricyclic antidepressants, gabapentinoids, NSAIDs, steroids, or muscle relaxants. These infusions should also be considered in situations when hyperalgesia appears to play a part in pain processing and perception.

Inpatient providers or care teams using ketamine infusions for the purpose of analgesia in severe refractory pain should be managing all opioid prescribing and pain management plans for the patient. Clear orders for monitoring of hemodynamic and respiratory status as well as dose titration should be established when infusion is initiated.

## CASE 1

**Acute Cancer Pain Crisis Refractory to Opioid Escalation**

A 27-year-old female with newly diagnosed stage IV Ewing’s sarcoma and diffuse osseous metastases to the axial and appendicular skeleton as well as pulmonary and splenic lesions was admitted to the hospital for uncontrolled pain. Imaging studies showed evidence of a mass displacing the right femoral neurovascular bundle anteriorly and laterally. She complained of pain (10/10 based on a verbal reporting scale [VRS]), with increased pain with any movement, and was noted to be tearful with frequent moaning. She demonstrated nociceptive bony pain, secondary myofascial pain related to immobility, as well as visceral abdominal pain and neuropathic pain in her extremities, particularly the right leg. She had previously been treated with gabapentin 1,800 mg/day in divided doses, without improvement in her pain and with the dose-limiting side effect of severe dizziness. Because of this experience, she was also unwilling to trial pregabalin.

On our initial visit, the patient was on a fentanyl transdermal patch at 100 μg/hr and was receiving hydromorphone and fentanyl doses intravenously, alternating every 30 to 60 minutes. A hydromorphone PCA was initiated, with a continuous basal rate and a PCA bolus dose every 10 minutes in addition to a nurse-administered bolus available every hour. Tizanidine (4–8 mg three times daily as needed) was added for muscle spasms, and nortriptyline (25 mg) was started once daily at night for neuropathic pain.

On hospital day 2, the patient complained of 8/10 pain increasing to 10/10 with movement and had received a total of 63 mg of IV hydromorphone in addition to the previously mentioned transdermal fentanyl, tizanidine, and nortriptyline. Given the lack of improvement despite significant opioid escalation and initiation of adjunct medications, a ketamine infusion was started at 0.1 mg/kg/hr (10 mg/hr), with rate adjustment parameters for the nursing staff to increase the rate by 2 mg/hr every 2 hours to a maximum of 20 mg/hr. In addition, the continuous basal rate and bolus rates of her hydromorphone PCA were increased. Over the next day, the ketamine infusion rate was increased slowly to 19 mg/hr.

On day 3, the patient’s pain improved to a pain score of 6/10, and she was able to sleep and move around in bed without severe pain. She expressed satisfaction with this improvement and felt that this level of pain was manageable for her. She did not demonstrate any dose-limiting side effects, and her hemodynamic parameters remained within normal limits throughout. At that time, the fentanyl transdermal patch was increased to 200 μg/hr, and the nortriptyline dose was increased to 75 mg at night.

Over the next 24 hours, the ketamine infusion was slowly decreased to 8 mg/hr and then discontinued. Oral hydromorphone was added for breakthrough pain, and her PCA was discontinued. The patient’s improved pain control was maintained with this combination of oral and transdermal medications and infrequent IV hydromorphone boluses. She was able to ambulate in the halls and participate with physical therapy. Continued adjustments were made over the next 24 hours to transition her off of IV hydromorphone.

She was discharged from the hospital on transdermal fentanyl 300 μg/hr for long-acting pain control, oral hydromorphone 8 mg every 4 hours for breakthrough pain, tizanidine 4–8 mg three times daily as needed, and nortriptyline 100 mg at night. At her first Supportive Oncology and Survivorship clinic visit 1 week after discharge, she continued to report manageable pain control on this regimen and requested no dose changes.

## CASE 2

**Uncontrolled Cancer Pain in the Setting of Hemodynamic Instability**

A 40-year-old male with advanced stage IV rectal cancer including liver and lymph node involvement with an extensive treatment and surgical history over several years was admitted to the intensive care unit (ICU) for renal failure, hypotension, and increasing abdominal pain.

This patient was well known to our service from the outpatient setting and a recent hospitalization for abdominal pain. His pain medications had been recently increased to a fentanyl transdermal patch at 125 μg/hr, with oxycodone at 15 mg every 3 hours as needed for breakthrough pain.

When he was admitted to the ICU, the fentanyl patch was decreased to 75 μg/hr, and the oxycodone was stopped due to concerns about somnolence and hypotension. Instead, he was started on oral hydromorphone at 4 mg every 2 hours as needed for moderate pain and hydromorphone at 0.2 mg IV every 2 hours as needed for severe pain. His renal failure improved with fluid resuscitation early in his hospital course, but he had persistent mild-to-moderate hypotension, without tachycardia, and severe left-sided abdominal and flank pain.

Our Supportive Oncology Service was consulted, and at our initial visit (day 1), he described his pain as 7/10 at best and 10/10 at worst, based on a VRS, with a tight pressure and squeezing quality to his abdomen and lower back. On exam, he exhibited ascites and anasarca, with significant scrotal edema. His previous 24-hour total doses of opioids included fentanyl patch at 75 μg/hr, oral hydromorphone at 48 mg, and IV hydromorphone at 0.2 mg. The fentanyl patch and hydromorphone doses were increased.

However, by day 2, the patient complained of being miserable, with pain 10/10 on a VRS. The oral morphine equivalent dose over the prior 24 hours was 580 mg. A hydromorphone PCA was instituted, and tizanidine at 2–4 mg three times daily as needed was started for muscle spasms. The patient felt the tizanidine was very helpful for pain, although he had notable worsening of hypotension following its administration, and this was discontinued. Given the patient’s persistent hemodynamic instability and uncontrolled pain, a ketamine infusion was started at 0.1 mg/kg/hr (7 mg/hr), with rate adjustment parameters for the nursing staff up to a maximum of 20 mg/hr.

On day 3, his pain was significantly better, rated at 5/10 typically and 7/10 at worst on a VRS, with no dose-limiting side effects or worsened hemodynamics with the ketamine infusion increased to 15 mg/hr. As the patient’s total 24-hour oral morphine equivalent dose totaled 855 mg, the transdermal fentanyl patch and hydromorphone PCA doses were increased.

By day 4, ketamine had been increased to 18 mg/hr without any ketamine-induced psychotomimetic effects, such as vivid dreams, hallucinations, or dysphoric mood, and with stable and improving hemodynamics. The oral morphine equivalent dose totaled 1,400 mg, and he felt that his pain was manageable overall; he was able to ambulate in the halls and visit with his children. At that time, the opioid doses were adjusted upward accordingly, and we started to titrate down the ketamine infusion.

On day 5, the ketamine infusion was decreased to 13 mg/hr, and oral hydromorphone was started to reduce the need for hydromorphone PCA. During his hospitalization, he was found to be experiencing rapid disease progression, and his ascites had accumulated to a point where his abdomen had become taut. He felt his pain was now controlled to a point that he could tolerate paracentesis, and 2 L of fluid was drained, with some improvement in pain but worsening in muscle spasms. As his hypotension had resolved, tizanidine at 4 mg three times daily as needed was reinitiated, without adverse effect. Gabapentin was also started at 300 mg three times daily for complaints of numbness and tingling to his left lower back and abdomen.

On day 6, he reported pain levels of 3 to 5/10 on a VRS, and the ketamine infusion was stopped. The oral morphine equivalent had decreased to 780 mg. After a goals-of-care discussion, the patient decided to transition to hospice care at home as soon as possible. He felt that his pain was well managed on his current medications, and he was discharged home on transdermal fentanyl, oral hydromorphone, hydromorphone PCA as needed, and adjuvant medications including tizanidine and gabapentin.

## DISCUSSION

In the setting of cancer, pain is a common and distressing symptom that can often be difficult to manage and is occasionally refractory to increasing opioids and adjunct medications. Ketamine is an NMDA-antagonist anesthetic agent with the potential to produce significant analgesia, and reports of its successful use for the treatment of cancer pain are found in the literature. However, with a dearth of high-quality randomized controlled trials of ketamine in this population, there is a need for further research to identify what type of cancer patients and which types of cancer pain are best treated with ketamine.

In the case presentations, ketamine infusions at subanesthetic doses helped to reduce acute pain crises and allowed improved response to opioid and adjuvant analgesic medications. Our ketamine infusion protocol is initiated at a weight-based dose of 0.1 mg/kg/hr and slowly titrated upward to a maximum of 20 mg/hr based on patient reports of comfort levels, with the goal of achieving pain control without psychotomimetic effects. At these low doses, we have found that dissociative side effects are rarely reported, and hemodynamic status is not significantly altered.

In our experience, in patients with advanced cancer and intractable pain, ketamine in subanesthetic doses can produce objective pain relief with few side effects. We have also found that it is most effective when used short term (3 to 6 days) as a complement to opioids and adjuvant medications such as neuropathic pain agents, muscle relaxants, steroids, and NSAIDs when appropriate.

## SUMMARY

Ketamine infusions at subanesthetic doses can help to interrupt and reverse pain crises involving complex cancer pain syndromes through antagonism of the NMDA receptors, which have been implicated in reduced analgesia in response to opioids (Kerr, Holahan, & Milch, 2011; Winegarden et al., 2015). Because the analgesia achieved by ketamine is not mediated by opioid receptors, it is a useful agent when there are difficulties with opioid responsiveness (Prommer, 2012). Psychotomimetic side effects are possible, although typically mild, and include impaired attention, memory, and judgment.

In the two case scenarios presented, we illustrated how ketamine infusions can be initiated in addition to combination opioid and adjuvant medication regimens for refractory pain crises, producing objective pain relief with few side effects within a short period. We have found that subanesthetic ketamine infusions are appropriate and beneficial for cancer patients receiving palliative care with complex pain syndromes (including nociceptive and neuropathic pathways) in situations where pain is unresponsive to opioid and adjunct escalation or further escalation is prevented by dose-limiting side effects. However, more research is needed to determine which patients with refractory pain would benefit most from the use of ketamine.
